# Root Metabolite Differences in Two Maize Varieties Under Lead (Pb) Stress

**DOI:** 10.3389/fpls.2021.656074

**Published:** 2021-11-23

**Authors:** Hanqian Zhang, Yuying Qin, Kai Huang, Fangdong Zhan, Ru Li, Jianjun Chen

**Affiliations:** ^1^College of Resource and Environment, Yunnan Agricultural University, Kunming, China; ^2^College of Plant Protection, Yunnan Agricultural University, Kunming, China; ^3^State Key Laboratory for Conservation and Utilization of Bio-Resources in Yunnan, Yunnan Agricultural University, Kunming, China

**Keywords:** *Zea mays*, maize varieties, metabolite, Pb stress, ultra-performance liquid chromatography–mass spectrometry

## Abstract

To assess root metabolic differences of maize varieties in their response to lead (Pb) stress, the lead-tolerant variety Huidan No. 4 and the lead-sensitive variety Ludan No. 8 were tested under Pb-free and Pb-stressed conditions. Changes in metabolites were measured using ultra-performance liquid chromatography–mass spectrometry. Pb stress changed the levels of the amino acids proline, glutamine, lysine, and arginine in both varieties, whereas glutamate and phenylalanine levels changed only in Huidan No. 4. Pb stress altered cystine, valine, methionine, and tryptophan levels only in Ludan No. 8. Therefore, the synthesis and decomposition of amino acids may affect the response of maize to Pb stress. The degree of change in differential metabolites for Huidan No. 4 was greater than that for Ludan No. 8. In cell wall subcellular components, increases in superoxide dismutase (SOD), peroxidases (PODs), and Pb concentrations were greater in Huidan No. 4 than in Ludan No. 8. Therefore, the greater Pb tolerance of Huidan No. 4 could be due to better sequestration of Pb in cell walls and more effective removal of reactive oxygen species (ROS) from the plant. The levels of certain metabolites only increased in Ludan No. 8, indicating that Pb-sensitive varieties may use different metabolic pathways to cope with Pb stress. Both varieties showed increased levels of some metabolites related to antioxidant protection and osmotic regulation. This study provides an understanding of maize Pb tolerance mechanisms and a basis for further development of tools for use in maize breeding.

## Introduction

Maize, an annual herbaceous plant in the genus *Zea* of the family Poaceae, is an important food and feed crop worldwide. It is also used as an industrial raw material and has the highest total output of any crop (Li, 2017). Heavy metal stress can affect the yield and quality of maize because these elements are highly toxic and difficult to detect and remove. For example, lead (Pb) is extremely harmful to human health. After entering the body, Pb and Pb-containing compounds can damage the nerves, kidneys, blood, bones, and immune and reproductive systems (Caito et al., 2017). According to a Chinese national soil survey report in 2014, 16.1% of soil samples exceeded the standard acceptable values for heavy metal pollutants; Pb ranked fifth, with 1.5% of soil samples exceeding allowable levels. Pb in the soil mainly enters the food chain *via* plant absorption, becoming hazardous to human health when consumed (Guan and Sun, 2014).

Pb stress can affect seed germination, root growth, photosynthesis, cell division, transpiration, and other plant functions (Meyers et al., 2010). Ahmad et al. (2011) found that Pb stress reduced maize photosynthesis and water use efficiency, enhancing transpiration efficiency. Plants can respond to environmental stress by changing their physiological functions and rebuilding metabolic networks (Obata and Fernie, 2012). Under Pb stress, plants synthesize various metabolites and signal molecules, such as antioxidants, osmotic adjustment substances, organic acids, and phenolic compounds (Sharma and Dietz, 2006). For example, phenolic content, including chlorogenic acid and rutin, increased in maize treated with Cd, Cu, and Pb (Kısa et al., 2016). The production of antioxidant enzymes such as guaiacol peroxidase, ascorbic peroxidase, catalase, and other antioxidant enzymes increased significantly in radish plants under Pb stress (Elbeltagi and Mohamed, 2010).

Non-targeted metabolomics is a comprehensive means of analyzing various metabolites to assess plant responses to stress; several recent studies have used this approach. Zhao et al. (2016) examined cucumber plants under nano-Cu stress and showed nano-Cu particles interfere in in the absorption of micronutrients. They also found that the main defense mechanisms in cucumber include the upregulation of amino acids to sequester nano-Cu, downregulation of citric acid to reduce the mobilization of Cu ions, ascorbic acid upregulation to combat reactive oxygen species (ROS), and upregulation of phenolic compounds to augment antioxidant systems. A gas chromatography–mass spectrometry analysis of radish roots to assess the metabolites and pathways involved in the stress response to lead revealed profound biochemical changes in the metabolism of glutathione, carbohydrates, and energy under Pb stress (Wang et al., 2016). Pb stress could have a greater impact on castor bean (*Ricinus communis* L.) roots than leaves, and the accumulation of malondialdehyde (MDA) and proline indicated the occurrence of lipid peroxidation and free radical scavenging mechanisms under lead stress (Pal et al., 2013). Dey et al. (2007) found that wheat grown in CdCl_2_ (0–200 μM) and Pb (NO_3_)_2_ (0–2000 μM) led to changes in the activity of different antioxidant enzymes.

Most previous plant metabolomics studies have compared responses between plants before and after stress. Few studies have examined the stress-response differences between varieties of the same species. Huidan No. 4 and Ludan No. 8 are maize varieties with different Pb tolerance levels. In this study, the metabolic responses of these varieties were studied under Pb stress using ultra-performance liquid chromatography–mass spectrometry (UPLC-MS) technology. This approach can reveal the comprehensive biochemical network of maize involved in response to Pb stress and ultimately improve maize food safety by breeding more tolerant varieties.

We hypothesize that the Pb tolerance mechanism of Huidan No. 4 involves the effective removal of ROS from the plant, and sequestration of Pb in cell walls.

## Materials and Methods

### Maize Varieties

The maize varieties “Huidan No. 4” and “Ludan No. 8” were used (Yu et al., 2014). Maize seeds were imbibed and incubated at 28°C for 3 days. Seedlings of the same growth stage were selected and transferred to Hoagland nutrient solution for 5 days. Five seedlings of each treatment were again selected for uniformity and planted in separate jars with Hoagland nutrient solution with and without Pb acetate [Pb (C_2_H_3_O_2_)_2_], and then placed in the greenhouse of Yunnan Agricultural University. Four treatments were established: Huidan No. 4 and Ludan No. 8 under Pb-free conditions, and each variety with 300 mg/L Pb acetate. The selection of this concentration of Pb acetate was based on the preliminary results that both maize varieties could survive throughout the experimental period (unpublished data). Five plants per treatment were used as replicates, and the plants were maintained for 14 days under Pb stress with the nutrient solutions renewed after 7 days.

### Analysis of Pb Concentration in Subcellular Components

Differential centrifugation was used to separate various cell components. Fresh root samples (200 mg) were weighed, ground, and homogenized with 20 mL of pre-cooled subcellular extraction solution (0.25 mmol/L sucrose, 50 mM Tris–HCl buffer at pH 7.5, 1 mmol/L dithioerythritol) at 4°C (Weigel and Jäger, 1979). The resulting extract was centrifuged at 300 rpm for 1 min to isolate the cell wall fraction from the pellet. The resulting supernatant was centrifuged again at 10,000 rpm for 20 min to separate the organelle fraction from the soluble fraction. Thereafter, each fraction was transferred into a flask, and 3 mL of nitric acid (68%) was added. After 12 h at room temperature, hydrogen peroxide (2 mL; 30%) was added. The flasks were placed in an oven at 130°C for 4 h and then allowed to cool to room temperature. The resulting digestion was diluted to 50 mL with double distilled H_2_O. After shaking, the Pb concentration was measured using a flame atomic absorption spectrophotometer (TAS-990, Beijing Puxi Instrument Factory, Beijing, China).

### Protein Extraction and Antioxidant Enzyme Activity Assay

Protein extraction was performed according to the manufacturer’s protocol (C500053, Sangon Biotech, Shanghai, China). The Coomassie brilliant blue G-250 staining method was used to determine the protein content of the enzyme (Bradford, 1976). The activities of the antioxidant enzymes superoxide dismutase (SOD) and peroxidase (POD) were determined using commercially available assay kits (Grace Ltd., Suzhou, China) following the manufacturer’s protocols and according to the method of Shi et al. (2013). The volume of reactive liquid was 800 μL. Enzyme activity was calculated as follows:


Total SOD activity (U/mg protein)=Blank OD value−measued OD valueBlank OD value÷50%  ×Total valume of reactive liquidSampling amount (mL)  ÷Concentration of the sample protein (mg protein / mL)POD activity (U/mg protein)=Measured OD value−blank OD value12×Chromatic light diameter (1 cm)  ×Total volume of reative liquid (mL)Sampling amount (μ L)  ÷Concentration of the sample protein (mg protein / mL)×1000

### Extraction of Root Metabolites

On the 14th day, 0.2 mg of root tissue from each experimental unit was transferred into a 15 mL tube with liquid nitrogen for homogenization. Thereafter, 2 mL of 100% methanol was added and sonicated for 30 min on ice. The resulting extract was evaporated to dryness, and the residue was resuspended in 200 μL methanol. The extract suspension was centrifuged for 10 min at 12,000 rpm at 4°C, and the supernatant was prepared for UPLC-MS examination. Quality control (QC) samples were prepared from the processed extracts by pooling 10 μL of each sample in this study set. Five replicates per treatment and four QC samples were prepared. Non-targeted metabolomics analysis of the samples using UPLC-MS was conducted at the Baitai Paike Company in Beijing.

### Ultra-Performance Liquid Chromatography–Mass Spectrometry Conditions

Ultra-performance liquid chromatography–mass spectrometry was performed on a TripleTOF 5600+ mass spectrophotometer (AB SCIEX^TM^, United States) equipped with a Turbo V electrospray ionization (ESI) source and a Shimadzu Nexera UHPLC LC-30A system. The chromatographic column was a Waters HSS T3 (150 mm × 3 mm, 1.8 μm). UPLC-MS conditions were as follows: The mobile phase of UPLC was composed of solvent A (acetonitrile) and solvent B (0.1% CH_3_COOH-H_2_O) at a constant flow rate of 0.3 mL/min. The initial mobile phase consisted of 0% A and 100% B, after which the gradient was changed to 50% A and 50% B at the 10th minute, then to 95% A and 5% B at the 13th minute, followed by 0% A and 100% B at the 14th minute until the endpoint at the 15th minute. The column temperature was maintained at 35°C, and the sample manager temperature was set at 4°C. Mass spectrophotometer parameters were as follows: both positive (5000 V) and negative (4500 V) ESI modes were performed to enhance metabolome coverage, the MASS scan range was 100–1500 m/z, and the scan model was window-based acquisition data-independent acquisition (DIA). The capillary temperature was maintained at 500°C throughout the run.

### Data Preprocessing

The raw UPLC-MS data were converted to the ABF format using the Reifycs Abf (Analysis Base File) Converter. The resulting data were imported to MS-DIAL version 3.82 (MS-DIAL: data-independent MS/MS deconvolution for comprehensive metabolome analysis). MS-DIAL software was used for data pre-processing, including peak extraction, denoising, deconvolution, peak alignment, and export of the 3D data matrix (raw data matrix) in CSV format (Tsugawa et al., 2015). The alignment was based on the m/z value and the retention time of the ion signals (retention time tolerance, 0.05 min; MS1 tolerance, 0.015 Da). The peak detection parameters were set with a minimum peak height of 1000 amplitude and 0.1 mass slice width. The deconvolution parameter sigma window value was set to 0.5. Metabolite identification was based on MS/MS with the setting as followed: retention time tolerance was 100 min, both MS1 and MS2 tolerances were 0.05 Da, and the identification score cut off was 80%. Since standards were not available, metabolites were annotated putatively by comparing their mass spectra to the mass spectra in the MassBank, Respect, and GNPS databases (14,951 records).

### Statistical Analysis

The relative peak area data set of metabolites was processed for multivariate analysis. Principal component analysis (PCA) was first used as an unsupervised method for data visualization and outlier identification. QC samples were tightly clustered together on the PCA score map (Supplementary Figure 1), indicating that the instrument and the mass spectrometry sequence process were stable and that the data were suitable for analysis. Thereafter, supervised regression modeling was performed on the dataset using partial least squares discriminant analysis (PLS-DA) to filter the potential biomarkers (MetaboAnalyst 4.0). Variable importance in projection (VIP) was estimated for metabolites with VIP < 0.5, which were removed from the final model. Differential metabolites were screened according to VIP values > 0.5, *P* ≤ 0.05, fold change (FC) ≥ 1.5, or FC ≤ 0.67. The data set of the relative peak areas of metabolites and antioxidant enzyme activity for the two maize varieties were processed using two-way ANOVA, followed by a mean separation test (Duncan’s Least Significant Difference) (*P* = 0.05) using SPSS software (version 21).

## Results

### Changes in Antioxidant Enzyme Activities

**FIGURE 1:**
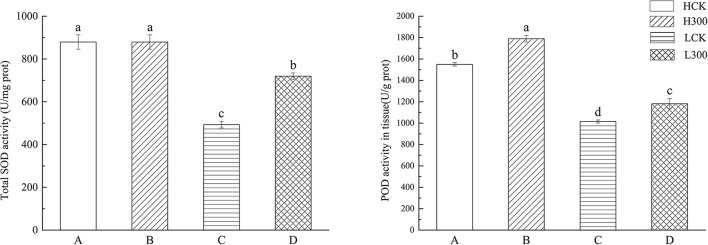
The difference of antioxidant enzyme activity between two varieties of maize. Huidan No. 4 lead-free treatment **(A)**, Huidan No. 4 exposed to lead **(B)**, Ludan No. 8 lead-free treatment **(C)**, and Ludan No. 8 exposed to lead **(D)**. The different letters indicate the antioxidant enzyme activity are significantly different among treatments (*P* < 0.05, Duncan’s Least Significant Difference).

Under Pb stress, SOD activity in Huidan No. 4 did not change significantly. In contrast, SOD activity in Ludan No. 8 increased by 45%. Compared with the control, POD activity increased by 15.49% in Huidan No. 4 and by 16.35% in Ludan No. 8 (Figure 1).

### Pb Content in Subcellular Components

**FIGURE 2:**
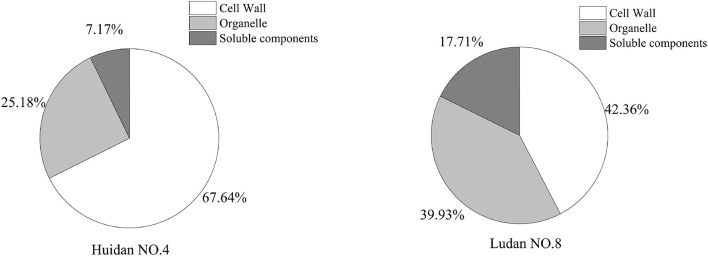
Subcellular distribution of lead in two maize varieties.

Under Pb stress, Pb accumulated in the three subcellular components of the maize roots, including cell walls, organelles, and the soluble component fraction. The Pb levels in the root cell walls of Huidan No. 4 (67.64%) and Ludan No. 8 (42.36%) were higher than those in the other subcellular components (Figure 2). The concentration of Pb in the root cell walls of Huidan No. 4 was higher than that of Ludan No. 8, whereas the concentration of Pb in organelles and soluble components was lower in Huidan No. 4.

### Metabolite Profiles for Huidan No. 4 and Ludan No. 8

**FIGURE 3:**
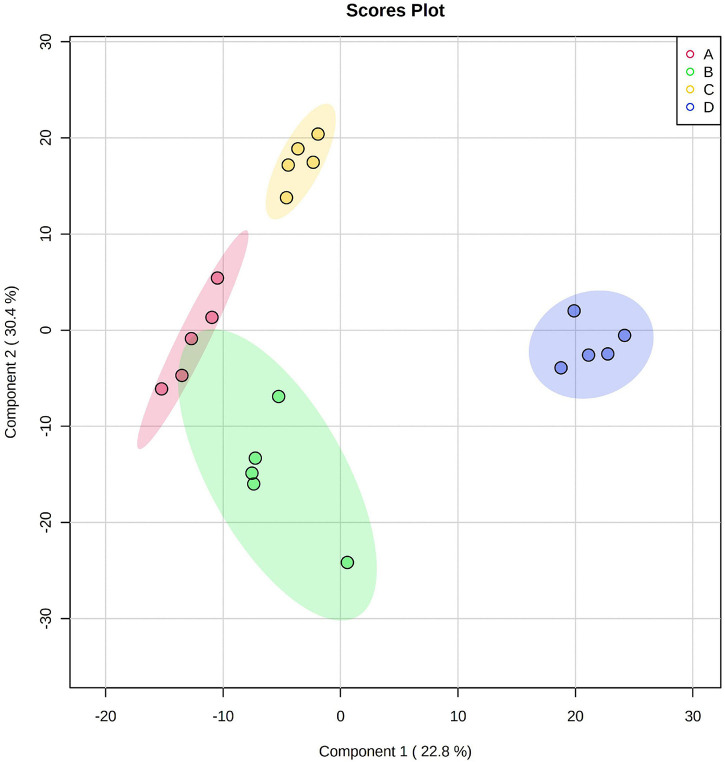
Partial least squares discriminant analysis of metabolic profiles in roots of two maize varieties. Huidan No. 4 lead-free treatment **(A)**, Huidan No. 4 exposed to lead **(B)**, Ludan No. 8 lead-free treatment **(C)**, and Ludan No. 8 exposed to lead **(D)**.

A total of 219 metabolite peak areas were compared among the different treatments. The PLS-DA demonstrated that the Pb tolerant variety Huidan No. 4 differed from the Pb-sensitive variety Ludan No. 8. Furthermore, Pb-treated maize was also separated from Pb-free maize, with 22.8 and 30.4% of variance explained by components 1 and 2, respectively (Figure 3). In Huidan No. 4 under Pb stress, 122 metabolites increased, 97 metabolites tended to decrease, and some metabolites showed a mixed pattern among replicates (Supplementary Figure 2). Under Pb stress, Ludan No. 8 showed an increasing trend for 152 metabolites and a decreasing trend for 67 metabolites. Similarly, some metabolites showed a mixed pattern among replicates (Supplementary Figure 3).

Differential metabolites were screened out according to VIP values > 0.5, FC ≥ 1.5, or FC ≤ 0.67, *P* ≤ 0.05. A total of 11 differential metabolites were screened in Huidan No. 4, of which eight increased and three decreased. A total of 17 metabolites in Ludan No. 8 were identified as being upregulated to varying degrees (Table 1 and Figure 3).

**TABLE 1:** Metabolites and their change fold in roots of Huidan No. 4 and Ludan No. 8 exposed to lead.

Metabolite	Fold change	
	Huidan No. 4	Ludan No. 8
Amino acids		
Proline	0.35^*b*^	2.11^*a*^
Glutamine	25.65^*a*^	4.95^*a*^
Lysine	40.73^*b*^	11.38^*a*^
Arginine	35.11^*b*^	13.09^*a*^
Glutamate	56.49	–
Phenylalanine	437.02	–
Cystine	–	26.03
Valine	–	4.34
Methionine	–	17.74
Tryptophan	–	6.72
Organic acids		
Malic acid	92.50^*a*^	77.52^*a*^
Oleic acid	0.30	–
Pyridine alkaloids		
Nicotinamde	–	5.99
Purines		
Adenine	–	22.09
Ribonucleosides		
Uridine	–	11.17
Adenosine	–	5.41
Deoxyribonucleotides		
2′-Deoxyguanosine	–	16.74
Vitamins and cofactors		
Riboflavin	–	9.40
NA		
Hypotaurine	151.28	–
8-Oxo-2-deoxyadenosine	240.93	–
Flavone base +2O, 1MeO, C-Hex	0.16	–
Guanosine-3′,5′-cyclic monophosphate	–	114.73
3-Methyladenine	–	5.63

*The different letters indicate the change fold of metabolite are significantly different between two maize varieties (P < 0.05, Duncan’s Least Significant Difference).*

Five differential metabolites (proline, glutamine, lysine, arginine, and malic acid) were the same in Huidan No. 4 and Ludan No. 8, and the remaining 18 differed (Table 1). The degree of change in differential metabolites for Huidan No. 4 was greater than that in Ludan No. 8. Among them, proline decreased in Huidan No. 4, although it increased in Ludan No. 8 (Table 1).

**FIGURE 4:**
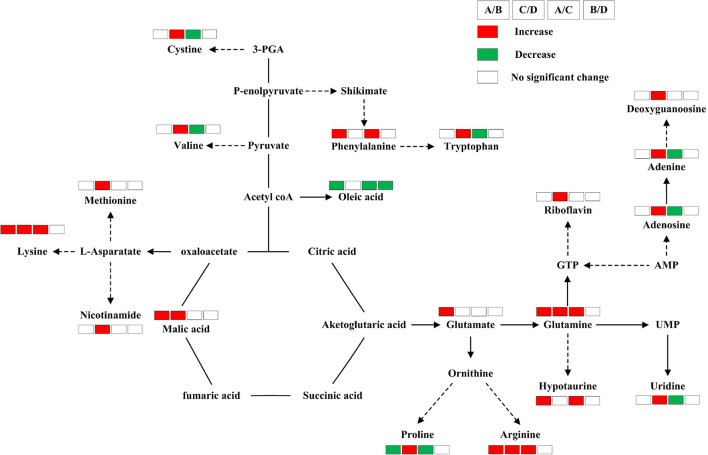
Metabolic changes involved in the primary pathways of Huidan No. 4 and Ludan No. 8 under lead exposure and lead-free treatments. Huidan No. 4 lead-free treatment **(A)**, Huidan No. 4 exposed to lead **(B)**, Ludan No. 8 lead-free treatment **(C)**, and Ludan No. 8 exposed to lead **(D)**. Metabolites with red boxes denote significant increases while with green ones denote significant decreases. The levels of significance was set at *P* < 0.05.

Among the 11 differential metabolites in Huidan No. 4 under Pb stress, six were amino acids and two were fatty acids. In contrast, the changes in metabolites in Ludan No. 8 were related to more diverse biological functions and metabolite pathways (Figure 4). Changes in Ludan No. 8 under Pb stress were observed for eight amino acids and two ribonucleosides. In addition, changes were also observed in the content of alkaloids, purines, deoxyribonucleotides, vitamins, and cofactors (Table 1).

### Amino Acid Changes

Under Pb stress, proline levels decreased in the Pb tolerant variety Huidan No. 4 but increased in the Pb-sensitive variety Ludan No. 8. Levels of glutamine, lysine, and arginine increased in both maize varieties under Pb stress. However, the degree of increase of these amino acids in Huidan No. 4 was much larger than that in Ludan No. 8 (Table 1). Glutamate and Phenylalanine levels increased significantly only in Huidan No. 4. The increase of phenylalanine was the largest among all the differential metabolites (Figure 4). In contrast, the levels of the other four amino acids (cystine, valine, methionine, and tryptophan) only changed significantly in Ludan No. 8.

### Organic Acid and Fatty Acid Changes

The levels of malic acid, an organic acid, significantly changed in both varieties. The level of oleic acid, a fatty acid, decreased significantly only in the Pb-tolerant variety Huidan No. 4 (Table 1).

### Other Metabolite Changes

In Huidan No. 4, the levels of hypotaurine and 8-Oxo-2-deoxyadenosine increased, whereas that of flavone base +2O, 1MeO, and C-Hex decreased. In Ludan No. 8, riboflavin, nicotinamide, guanosine 3,5-cyclic-phosphate (cGMP), and adenine levels increased significantly (Table 1).

## Discussion

### Antioxidases in Huidan No. 4 and Ludan No. 8

With respect to antioxidases, the results of this study were consistent with previous findings that Pb stress in maize induces the production of free radicals and ROS, such as singlet oxygen, superoxide free radicals, hydrogen peroxide, and hydroxyl radicals, thereby interfering with redox homeostasis (Gupta et al., 2009). Furthermore, levels of both SOD and POD in the Pb-tolerant variety Huidan No. 4 were much higher than those in the Pb-sensitive Ludan No. 8. SOD is an important antioxidant enzyme that scavenges superoxide and produces H_2_O_2_ and O_2;_ H_2_O_2_ is toxic and can be detoxified by POD (Noctor and Foyer, 1998). The higher concentrations of SOD and POD in Huidan No. 4 could enhance heavy metal detoxification and contribute to the Pb stress tolerance of this maize variety.

### Pb Sequestration in Subcellular Components of Maize Root

In this study, the analysis of the subcellular components of maize roots showed higher levels of Pb in the root cell walls of Huidan No. 4. Kumar et al. (1995) previously demonstrated that some plant species can stabilize metals in their roots and translocate low quantities of metals to the above-ground organs. Using X-ray spectroscopy-based techniques, it has been found that the lingo-cellulosic subcellular structure constituting the cell wall of the European walnut (*Juglans regia*) root could sequester Pb (Marmiroli et al., 2005). Our results are consistent with those of the previous studies.

### Differential Amino Acids in Huidan No. 4 and Ludan No. 8

In this study, most of the differential metabolites produced by the two maize varieties under Pb stress were amino acids. The mechanism of Pb resistance in plants may be related to osmotic adjustment, ion transport and balance, and antioxidant protection. Kaur et al. (2015) have reported that Pb stress induces a large release of active oxygen from maize root cells. Massive accumulation of ROS can disturb the redox balance of cells and damage biomolecules, such as membrane lipids, proteins, and nucleic acids. They can also cause cell death and reduce crop biomass and yield (Czarnockaa and Karpiński, 2018). To cope with ROS stress, plants produce various metabolites to eliminate active oxygen or the negative effects of active oxygen. Amino acids are components of plants and important plant metabolites. They have multiple functions, such as active oxygen elimination, signal transduction, cell osmotic adjustment, mineral nutrient absorption, and heavy metal detoxification (Singh et al., 2016). Amino acid metabolism is closely related to carbohydrate metabolism, the citric acid cycle, nitrogen metabolism, and secondary compound metabolism (Pratelli and Pilot, 2014). Our results support previous findings that amino acids are important in the response of maize to Pb stress.

This study found that under Pb stress, proline levels decreased, and the level of POD increased significantly in the Pb-tolerant variety Huidan No. 4. In contrast to the response of Huidan No. 4, proline increased in the Pb-sensitive variety Ludan No. 8. POD in Ludan No. 8 also increased significantly. Proline can act as a penetrant, free radical scavenger, macromolecular stabilizer, and metal chelating agent under heavy metal stress (Alia, 1991). In addition, proline can also act as a signaling molecule to change the transcription level of stress-related antioxidant enzyme genes, thereby increasing the level of plant antioxidant enzymes (Carvalho et al., 2013). It is possible that the significant differences in proline responses in the two maize varieties could be related to their different levels of Pb tolerance.

The levels of three amino acids, lysine, arginine, and glutamine, increased in both maize varieties under Pb stress. The increases in the Pb tolerant variety Huidan No. 4 were much larger than those in the Pb-sensitive variety Ludan No. 8 (Table 1). Differences in the metabolism of maize and vetiver (*Chrysopogon zizanioides*) under Pb stress have indicated that amino acids could play an important role in Pb tolerance (Pidatala et al., 2018). Our findings are consistent with these conclusions.

Arginine levels increased 35.11-fold in Huidan No. 4 and 13.09-fold in Ludan No. 8, indicating that accumulated arginine in maize roots may help plants withstand Pb stress. Arginine is a precursor for protein synthesis, as well as for amino acids, polyamines, and nitric oxide, which are important signaling molecules involved in plant development and anti-stress processes (Brauc et al., 2012). Among the 21 amino acids, arginine has the highest nitrogen-to-carbon ratio and is the most suitable storage form for organic nitrogen (Winter et al., 2015). In addition, arginine metabolism plays a key role in the distribution and circulation of nitrogen in plants (Slocum, 2005). Studies have shown that accumulated arginine and its catabolites can help plants cope with various abiotic and biotic stresses, such as water stress (Nasibi et al., 2011), drought (Desikan et al., 2003), low temperature (Ma et al., 2005), pathogens (Xu and Zhu, 2005), and salinity (Kakkar et al., 2000).

The content of lysine in both maize varieties increased by 40.73-fold in Huidan No. 4 and 11.38-fold in Ludan No. 8. Lysine is a nutritionally essential amino acid. Catabolism in plants is a super-regulated metabolic pathway that breaks down lysine into glutamic acid and acetyl-coenzyme A, which has multiple developmental and stress-associated functions (Galili et al., 2001; Stepansky et al., 2001, 2006). Thus, the enormous increase in lysine levels in Huidan No. 4 could be related to its mechanism of Pb tolerance.

Glutamate levels increased by 56.49-fold in Huidan No. 4, whereas there was no significant change in its levels in Ludan No. 8 (Figure 3). One of the main carbon and nitrogen assimilation and partitioning pathways in plants converts amino acid glutamate to ornithine, arginine, proline, polyamines, and intermediates such as nitric oxide and gamma-aminobutyric acid. This network has a wide range of physiological functions in abiotic and biotic stress responses (Bagni and Tassoni, 2001; Subramanian et al., 2015; Majumdar et al., 2016). Therefore, the significant increase in glutamate in Huidan No. 4 could contribute to the Pb stress tolerance of the variety.

Glutamate can be condensed with NH_3_ by glutamine synthetase to form glutamine, which participates in the metabolism of nitrogenous compounds in plants (Huang et al., 2010). The synthesis of glutamine is the only reaction that allows the assimilation of inorganic nitrogen into organic molecules. Thus, the pathways for the synthesis of all other nitrogen-containing compounds are linked to either glutamine or its sister metabolites at some point (Bernard and Habash, 2009). The ability of glutamine to monitor cellular nitrogen status is essential for the maintenance of plant metabolic homeostasis and growth (Chellamuthu et al., 2014). The increase in glutamine levels in Huidan No. 4 was much higher than that in Ludan No. 8, indicating that Huidan No. 4 could better maintain metabolic homeostasis than Ludan No. 8 under Pb stress.

Phenylalanine levels increased significantly only in Huidan No. 4; the increase was the largest among all the differential metabolites (Figure 4). Phenylalanine is one of the key metabolites in the shikimate pathway. In vascular plants, approximately 30% of the photosynthetic fixed carbon leads to the biosynthesis of lignin through phenylalanine (Boerjan et al., 2006). Plant cell walls can absorb heavy metals and prevent heavy metals from entering the cell. Cell walls are the first barrier to heavy metal accumulation in plants. Cell wall thickening is a general defense strategy for plants against heavy metals (Krzeslowska et al., 2016). Kopittke et al. (2008) have suggested that the adsorption of Pb by the cell wall is an important mechanism for Pb resistance in *Brachiaria decumbens*. Lignin is a key structural component of plant cell walls. The enhancement of phenylalanine levels in Huidan No. 4 could be related to its greater Pb stress tolerance compared to Ludan No. 8, as phenylalanine plays an important role in lignin biosynthesis and thus cell wall thickening. Simultaneously, the subcellular Pb concentration of Pb in the root cell walls of Huidan No. 4 was far greater than that of Ludan No. 8. Thus, the Pb-tolerant maize variety Huidan No. 4 had a greater ability to sequester Pb in its cell walls compared to the sensitive variety Ludan No. 8.

Cystine, valine, methionine, and tryptophan levels only changed significantly in Ludan No. 8. Cystine and valine increase in wheat under drought stress (Bowne et al., 2012) and in tobacco under salt stress (Zhang et al., 2011). However, there are no previous reports of the contents of these two amino acids increasing under heavy metal stress akin to the Pb stress imposed in this study. These amino acids may play a role in the response to water deficit caused by Pb stress. Zemanová et al. (2014) have shown that methionine and tryptophan content changes to varying degrees in plants under cadmium stress. Their results indicated that tryptophan might participate in cadmium resistance by reducing oxidative damage. Furthermore, methionine is related to plant defenses. *S*-adenosyl-L-methionine, metabolite produced by the methionine metabolic pathway plays roles under NaCl stress, ABA treatment, and oxidation and high-temperature stresses (She et al., 2015). The SOD and POD concentrations in Ludan No. 8 increased significantly; therefore, the increased tryptophan and methionine levels in Ludan No. 8 might help alleviate the oxidative damage caused by Pb stress.

### Organic Acid Content in Huidan No. 4 and Ludan No. 8

Malic acid levels increased significantly by 92.50-fold in Huidan No. 4 and 77.52-fold in Ludan No. 8. Malic acid is a low-molecular-weight organic acid mainly involved in regulating plant growth, stomatal opening, and the steady-state of nutrients (Finkemeier and Sweetlove, 2009). Malic acid plays a crucial role in the detoxification of heavy metals in maize leaves (Dresler et al., 2014). In addition, it can reduce cadmium-induced phytotoxicity and oxidative damage by regulating enzymes and non-enzymatic antioxidants under cadmium stress (Guo et al., 2017). Malic acid levels changed significantly in both varieties, indicating that it might contribute to Pb stress reduction.

### Fatty Acid Content in Huidan No. 4 and Ludan No. 8

Compared to the Pb-sensitive variety Ludan No. 8, oleic acid levels decreased significantly in Huidan No. 4. Fatty acid decomposition provides cellular energy and thus may participate in the process of the plant stress response (Masterson and Wood, 2001; Xu et al., 2008). The decrease in oleic acid in Huidan No. 4 under Pb stress might be due to the suppression of energy production caused by plant cell damage from Pb.

### Changes Specific to Huidan No. 4

In Huidan No. 4, hypotaurine and 8-Oxo-2-deoxyadenosine levels increased 151.28- and 240.93-fold, respectively, whereas that of flavone base +2O, 1MeO, C-Hex decreased 5.5-fold. Hypotaurine can eliminate H_2_S, a phytotoxin produced by plants under adverse conditions (Valivand et al., 2019). The function of 8-Oxo-2-deoxyadenosine is yet unknown. Therefore, further studies are needed to understand its function.

### Changes Specific to Ludan No. 8

Riboflavin, nicotinamide, guanosine 3,5-cyclic-phosphate (cGMP), and adenine levels increased significantly in Ludan No. 8. Pb stress can cause the accumulation of ROS that damage the cells. Riboflavin is a vitamin required for normal growth and development in plants; it can mediate the production of ROS to increase plant tolerance to adversity (Deng et al., 2014). Nicotinamide is a precursor of nicotinamide adenine dinucleotide, which plays a rate-limiting role in the generation and removal of ROS in Arabidopsis, thus protecting cells from ROS (Jander and Joshi, 2009). Nicotinamide affects maize lipid peroxidation under salt stress (Hassanein et al., 2009). Arabidopsis cGMP increases rapidly under salt and osmotic stress (Donaldson et al., 2004). Endogenous accumulation of cGMP can maintain ion balance in Arabidopsis (Li et al., 2014). Adenine in cells can function as a signaling molecule in the regulation of abiotic stress tolerance and plant growth (Sukrong et al., 2012). In this study, the levels of metabolites belonging to different biological functions changed much more in Ludan No. 8 compared to Huidan No. 4. This result might indicate that more metabolic pathways in the Pb-sensitive variety were destabilized under Pb stress.

## Conclusion

Few studies have compared the metabolic characteristics of two varieties of the same species under Pb stress. The two varieties studied herein shared some metabolites involved in responding to Pb stress, such as proline, glutamine, lysine, arginine, and malic acid. However, Huidan No. 4 showed a higher cumulative compound content. Phenylalanine, hypotaurine, and 8-Oxo-2-deoxyadenosine were significantly accumulated only in Huidan No. 4. We hypothesized that the higher Pb tolerance of Huidan No. 4 might be related to the sequestration of Pb in cell walls because phenylalanine is related to cell wall synthesis. The higher increase in Pb concentration in the cell wall subcellular component in Huidan No. 4 supports this hypothesis. SOD and POD levels increased more in Huidan No. 4 than in Ludan No. 8, indicating that Huidan No. 4 effectively removes active oxygen from the plant. For Ludan No. 8, a broader spectrum of metabolites with different biological functions changed. Moreover, different pathways were used in response to Pb stress, including cGMP, adenine, cysteine, valine, methionine, tryptophan, nicotinamide, uridine, and riboflavin. Riboflavin and nicotinamide are active oxygen scavengers, and cGMP and adenine can be generated in response to osmotic stress caused by Pb. In summary, Huidan No. 4 and Ludan No. 8 used some common metabolic pathways to alleviate Pb damage.

## Data Availability Statement

The raw data supporting the conclusions of this article will be made available by the authors, without undue reservation.

## Author Contributions

HZ: formal analysis, investigation, data curation, writing original draft preparation, and visualization. YQ and KH: investigation. FZ: resources and methodology. RL: formal analysis, data curation, and review and editing. JC: conceptualization, methodology, supervision, project administration, and funding acquisition. All authors contributed to the article and approved the submitted version.

## Conflict of Interest

The authors declare that the research was conducted in the absence of any commercial or financial relationships that could be construed as a potential conflict of interest.

## Publisher’s Note

All claims expressed in this article are solely those of the authors and do not necessarily represent those of their affiliated organizations, or those of the publisher, the editors and the reviewers. Any product that may be evaluated in this article, or claim that may be made by its manufacturer, is not guaranteed or endorsed by the publisher.

## Supplementary Material

Supplementary Figure 1 Quality control sample evaluation: the score plot of the PCA model. Huidan No. 4 lead-free treatment (1), Huidan No. 4 exposed to lead (2), Ludan No. 8 lead-free treatment (3), and Ludan No. 8 exposed to lead (4).

Supplementary Figure 2 Heatmap analysis of metabolic profiles in roots of Huidan No. 4 exposed to lead and lead-free treatment. Huidan No. 4 lead-free treatment (HCK), Huidan No. 4 exposed to lead (H300).

Supplementary Figure 3 Heatmap analysis of metabolic profiles in roots of Ludan No. 8 exposed to lead and lead-free treatment. Ludan No. 8 lead-free treatment (LCK), Ludan No. 8 exposed to lead (L300).
