# Supplementary figures and images for: Root Metabolite Differences in Two Maize Varieties Under Lead (Pb) Stress
Source: Front Plant Sci. 2021 Nov 23;12:656074. doi: 10.3389/fpls.2021.656074 (PMC8649664; doi:10.3389/fpls.2021.656074)

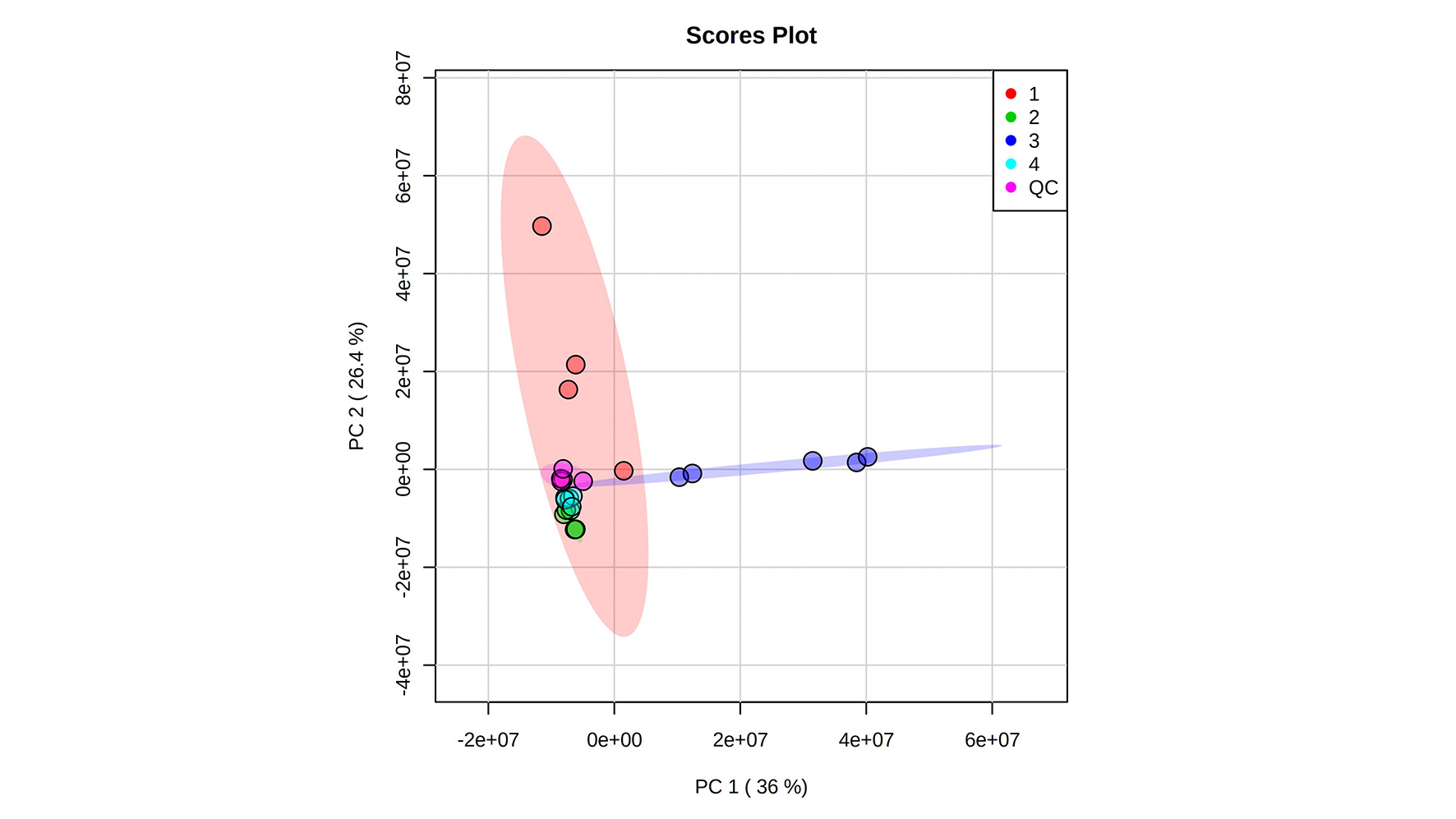

Supplement: Supplementary Figure 1 — Quality control sample evaluation: the score plot of the PCA model. Huidan No. 4 lead-free treatment (1), Huidan No. 4 exposed to lead (2), Ludan No. 8 lead-free treatment (3), and Ludan No. 8 exposed to lead (4). [file Image_1.JPEG]

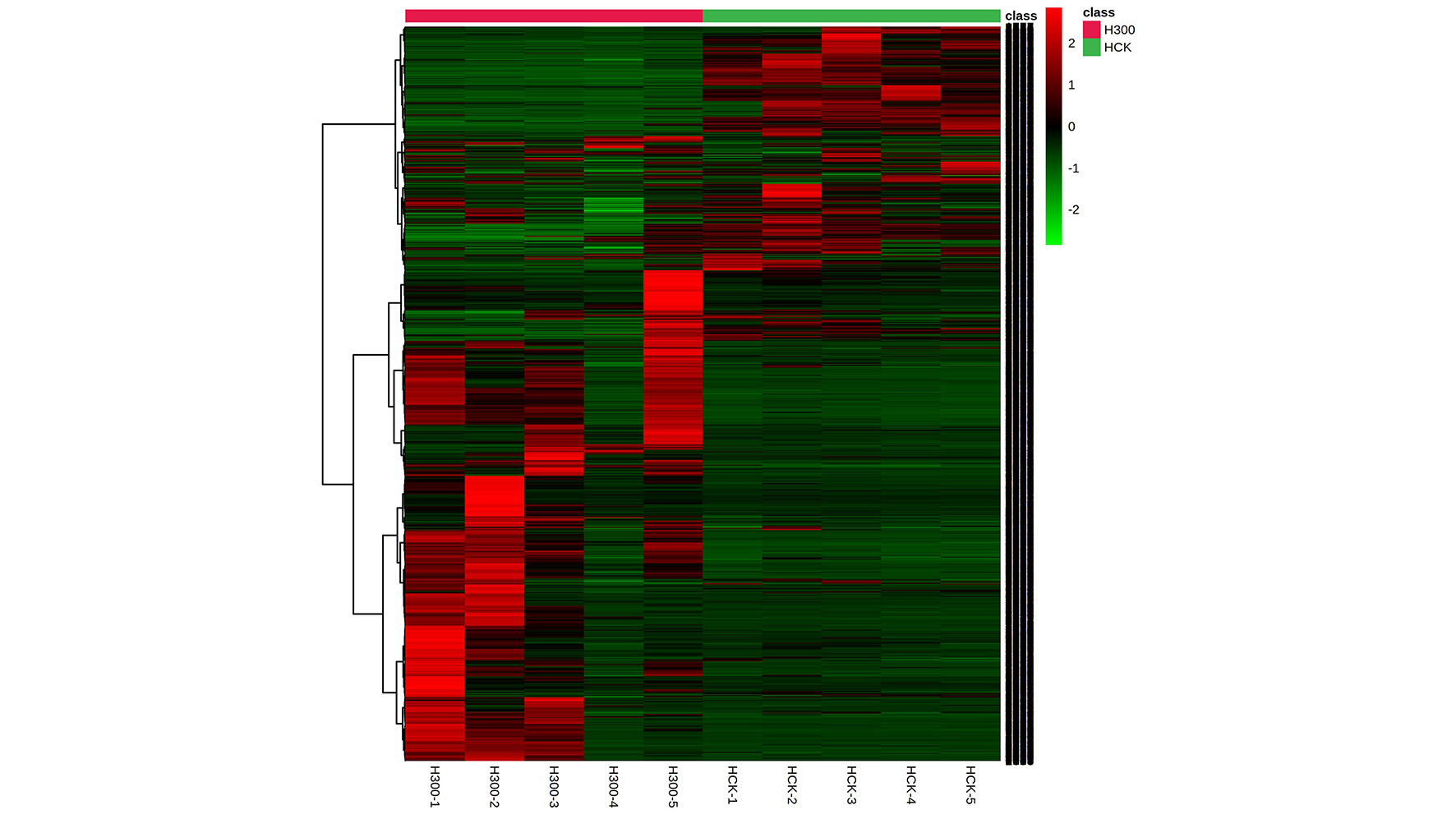

Supplement: Supplementary Figure 2 — Heatmap analysis of metabolic profiles in roots of Huidan No. 4 exposed to lead and lead-free treatment. Huidan No. 4 lead-free treatment (HCK), Huidan No. 4 exposed to lead (H300). [file Image_2.JPEG]

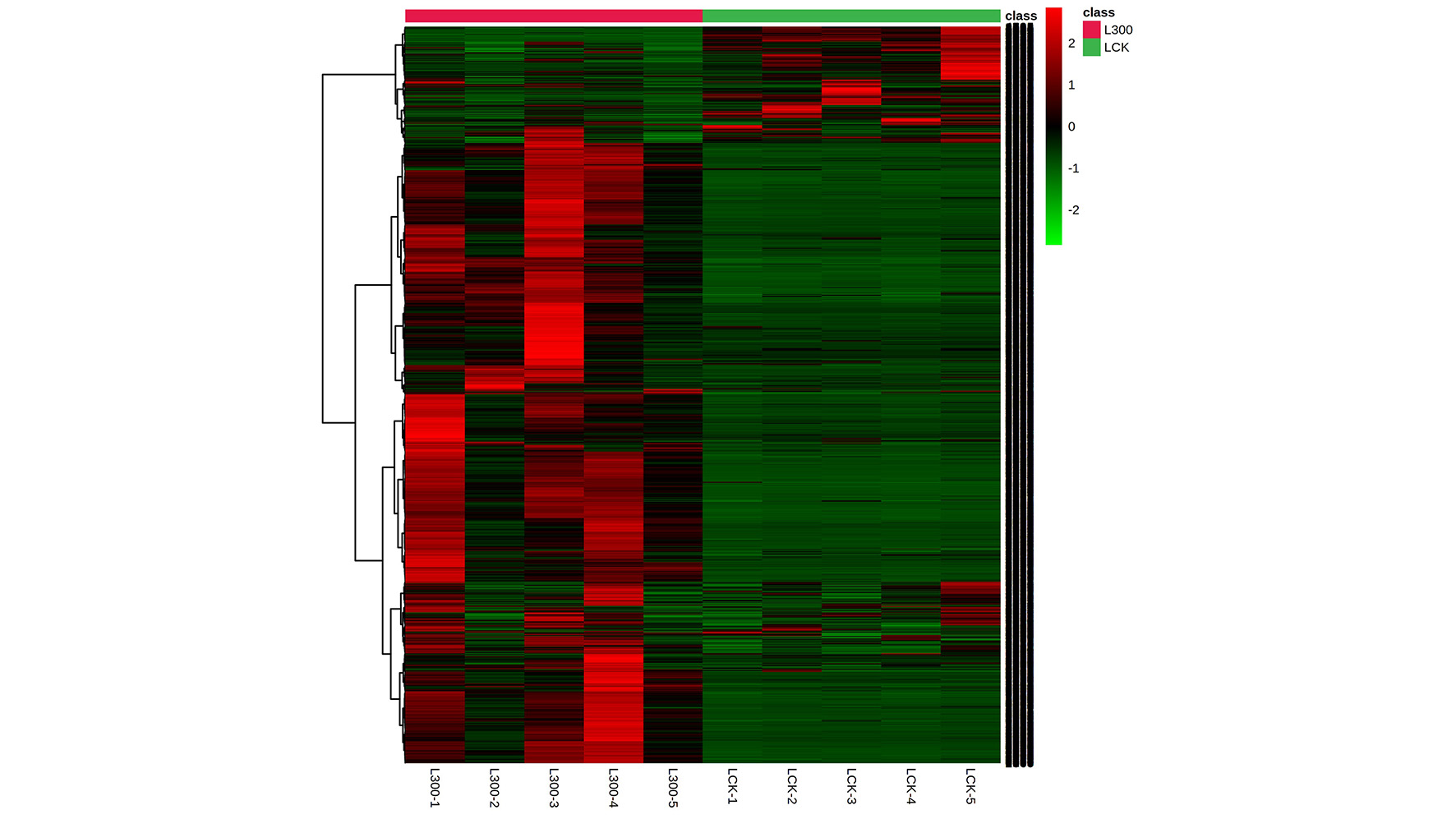

Supplement: Supplementary Figure 3 — Heatmap analysis of metabolic profiles in roots of Ludan No. 8 exposed to lead and lead-free treatment. Ludan No. 8 lead-free treatment (LCK), Ludan No. 8 exposed to lead (L300). [file Image_3.JPEG]
