# Supplementary material for: Root Metabolite Differences in Two Maize Varieties Under Lead (Pb) Stress
Source: Front Plant Sci. 2021 Nov 23;12:656074. doi: 10.3389/fpls.2021.656074 (PMC8649664; doi:10.3389/fpls.2021.656074)
Supplement: Supplementary file 4 [file Data_Sheet_1.docx]

Table S1. Characteristics of all differential metabolites

| metabolite name | retention time | accurate mass | molecular ion type | chemical molecular formula | identification fragment |  |
| --- | --- | --- | --- | --- | --- | --- |
|  |  |  |  |  |  |  |
| Proline | 0.92 | 116.0694 | [M+H]+ | C5H9NO2 | 70.07057:125 70.07175:125 70.07411:125 70.09892:125 116.07334:125 |  |
| Glutamine | 0.92 | 147.0749 | [M+H]+ | C5H10N2O3 | 61.46144:297 84.04622:2623 85.02947:252 88.04266:126 101.07344:257 102.05876:390 102.49965:273 119.09516:272 130.05211:1858 |  |
| Lysine | 0.81 | 147.1127 | [M+H]+ | C6H14N2O2 | 69.07423:287 84.08244:1553 84.10184:304 85.81575:127 130.08594:513 132.88298:127 |  |
| Arginine | 0.9 | 175.1166 | [M+H]+ | C6H14N4O2 | 74.03329:126 116.06921:252 141.08366:126 158.11877:126 175.11871:505 |  |
| Glutamate | 0.95 | 148.0604 | [M+H]+ | C5H9NO4 | 57.05713:268 74.02475:272 84.04488:1192 84.4502:274 85.05285:519 102.05872:397 107.05849:258 130.05048:385 131.11613:126 |  |
| Phenylalanine | 3.27 | 166.0485 | [M+H]+ | C9H11NO2 | 120.04692:259 166.05351:388 |  |
| Cystine | 6.02 | 241.0467 | [M+H]+ | C6H12N2O4S2 | 241.04411:383 |  |
| Valine | 1 | 118.086 | [M+H]+ | C5H11NO2 | 53.04825:139 55.05731:1260 55.06987:556 56.06264:257 57.06534:261 59.05511:268 72.08183:1333 |  |
| Methionine | 1.39 | 150.0561 | [M+H]+ | C5H11NO2S | 56.0534:252 56.06397:126 56.06502:126 56.06713:126 56.09989:126 61.01092:126 102.05302:126 104.04946:126 104.05953:126 133.03911:126 133.05539:126 150.02893:126 |  |
| Tryptophan | 3.1 | 205.0973 | [M+H]+ | C11H12N2O2 | 104.58089:263 115.05576:1063 117.07879:558 118.06548:4139 118.75941:271 128.06436:265 130.06784:1213 131.36955:273 132.08202:1110 133.71819:271 142.06363:1210 143.07266:1125 144.08189:2107 145.59303:345 146.06154:15705 146.27304:727 146.8931:655 149.02448:510 157.07697:263 159.09206:1820 170.05853:807 170.09349:616 172.49945:340 188.07477:7046 196.00421:126 |  |
| Malic acid | 0.99 | 133.0145 | [M-H]- | C4H6O5 | 71.01436:453 72.99818:63 114.99495:194 115.00251:403 133.01216:260 |  |
| Oleic acid | 12.71 | 281.2476 | [M-H]- | C18H34O2 | 281.24692:929 |  |
| Nicotinamide | 1.4 | 123.0553 | [M+H]+ | C6H6N2O | 78.04072:258 80.05516:696 93.04114:264 96.066:126 108.09125:261 123.05166:533 123.05479:811 |  |
| Adenine | 1.76 | 136.0616 | [M+H]+ | C5H5N5 | 119.03641:257 136.01534:257 136.02686:515 |  |
| Uridine | 1.47 | 245.0762 | [M+H]+ | C9H12N2O6 | 59.05181:259 69.05386:259 73.03235:259 73.04561:129 103.04692:536 113.03968:2943 116.1751:142 131.06068:382 133.05646:382 |  |
| Adenosine | 1.01 | 268.1029 | [M+H]+ | C10H13N5O4 | 136.05867:655 268.09512:252 |  |
| 2'-Deoxyguanosine | 2.16 | 268.103 | [M+H]+ | C10H13N5O4 | 110.04175:126 117.07848:260 135.02777:523 137.4104:295 152.0602:5241 152.55817:252 |  |
| Riboflavin | 3.57 | 377.1435 | [M+H]+ | C17H20N4O6 | 243.10265:378 377.14804:256 |  |
| Hypotaurine | 0.77 | 110.008 | [M+H]+ | C2H7NO2S | 66.04793:126 68.06036:126 68.98999:126 68.99351:126 68.99585:126 69.00523:126 72.05244:126 72.05363:126 73.0619:126 75.53901:126 76.20876:126 77.51614:126 78.03872:126 78.05617:126 78.0649:126 80.94807:126 82.01782:126 83.98564:126 87.00282:126 87.04494:126 |  |
| 8-oxo-2-deoxyadenosine | 2.26 | 268.1042 | [M+H]+ | C10H13N5O4 | 85.65775:126 117.0533:382 119.04755:261 135.03032:956 135.04671:413 136.06677:396 139.11003:300 151.57101:610 152.05948:9003 152.81372:252 159.08199:126 |  |
| Flavone base + 2O, 1MeO, C-Hex | 6.36 | 447.1299 | [M+H]+ | C22H22O10 | 149.0192:129 149.05193:266 171.13826:126 193.05058:802 193.07214:406 223.08038:278 237.07423:1903 240.71581:256 327.03064:270 341.07016:782 343.01926:270 361.03891:531 367.0119:258 385.08371:830 385.10031:863 387.46277:265 424.2052:132 429.12723:2038 431.09052:526 |  |
| guanosine-3',5'-cyclic monophosphate | 1.45 | 346.0555 | [M+H]+ | C10H12N5O7P | 135.03474:529 152.04849:293 152.05893:1644 152.08676:263 |  |
| 3-methyladenine | 4.23 | 150.0529 | [M+H]+ | C6H7N5 | 150.04749:128 |  |

Table S2. Peak area of each treatment for Huidan No. 4

| metabolite name | HCK | | | | | H300 | | | | | QC | | | | |
| --- | --- | --- | --- | --- | --- | --- | --- | --- | --- | --- | --- | --- | --- | --- | --- |
|  | HCK-1 | HCK-2 | HCK-3 | HCK-4 | HCK-5 | H300-1 | H300-2 | H300-3 | H300-4 | H300-5 | QC-1 | QC-2 | QC-3 | QC-4 |  |
| Proline | 43801 | 78325 | 93680 | 43130 | 54015 | 3497 | 3303 | 1599 | 3024 | 98210 | 218794 | 189279 | 2128 | 147965 |  |
| Glutamine | 9053 | 5790 | 6713 | 2097 | 4959 | 265010 | 86654 | 294259 | 73203 | 14715 | 42706 | 20987 | 114638 | 127154 |  |
| Lysine | 1350 | 1463 | 268 | 1140 | 2500 | 95474 | 73970 | 85142 | 14670 | 4472 | 2148 | 2179 | 27236 | 66506 |  |
| Arginine | 1876 | 1477 | 1405 | 1213 | 3691 | 105632 | 109358 | 121196 | 1688 | 1337 | 6085 | 3218 | 2328 | 104995 |  |
| Glutamate | 832 | 2352 | 1313 | 3238 | 2083 | 245341 | 86105 | 194980 | 3799 | 24410 | 6445 | 21329 | 118650 | 162113 |  |
| Phenylalanine | 4156 | 6950 | 6867 | 4559 | 7969 | 535338 | 604553 | 1107149 | 1379589 | 1375525 | 985136 | 349118 | 974240 | 299039 |  |
| D-(+)-Malic acid | 1104 | 3692 | 2291 | 1730 | 1753 | 238440 | 247463 | 209598 | 71 | 282195 | 127640 | 169677 | 235348 | 106801 |  |
| Oleic acid | 92154 | 97745 | 227802 | 83453 | 107114 | 69236 | 34830 | 38888 | 3987 | 33487 | 119535 | 131534 | 125444 | 122027 |  |
| HYPOTAURINE | 1884 | 2389 | 1395 | 1676 | 1433 | 445328 | 575139 | 282846 | 5384 | 19110 | 3070 | 2752 | 146173 | 331913 |  |
| 8-Oxo-2-deoxyadenosine | 624 | 683 | 647 | 324 | 427 | 36400 | 42255 | 231821 | 30784 | 310449 | 59861 | 213977 | 6012 | 32446 |  |
| Flavone base + 2O, 1MeO, C-Hex | 10715 | 101067 | 121880 | 140900 | 122348 | 24778 | 16809 | 15376 | 10400 | 14153 | 7965 | 114388 | 17351 | 97780 |  |

Table S3. Peak area of each treatment for Ludan No. 8

| metabolite name | LCK | | | | | L300 | | | | | QC | | | |
| --- | --- | --- | --- | --- | --- | --- | --- | --- | --- | --- | --- | --- | --- | --- |
|  | LCK-1 | LCK-2 | LCK-3 | LCK-4 | LCK-5 | L300-1 | L300-2 | L300-3 | L300-4 | L300-5 | QC1 | QC2 | QC3 | QC4 |
| Proline | 69842 | 89024 | 91567 | 54648 | 92456 | 109085 | 250136 | 295945 | 110602 | 73756 | 218794 | 189279 | 2128 | 147965 |
| Glutamine | 23826 | 50258 | 43056 | 30415 | 67507 | 222557 | 154778 | 298013 | 182647 | 206352 | 42706 | 20987 | 114638 | 127154 |
| Lysine | 6766 | 8772 | 15970 | 5620 | 7830 | 75743 | 149461 | 145470 | 66688 | 74329 | 2148 | 2179 | 27236 | 66506 |
| Arginine | 14291 | 2182 | 23075 | 9166 | 3308 | 96884 | 224934 | 198070 | 84043 | 76859 | 6085 | 3218 | 2328 | 104995 |
| L-Cystine | 4468 | 3335 | 4405 | 4870 | 5362 | 130617 | 103171 | 132949 | 136020 | 81396 | 158 | 85670 | 326 | 377 |
| Valine | 95506 | 94492 | 255114 | 72829 | 91439 | 474862 | 577034 | 783423 | 483490 | 328209 | 5570 | 336220 | 6960 | 1736 |
| Methionine | 3472 | 6027 | 6530 | 4457 | 4493 | 62595 | 106171 | 132596 | 78528 | 63215 | 26405 | 44080 | 6434 | 2237 |
| Tryptophan | 251532 | 172035 | 437945 | 352239 | 293932 | 1544555 | 2091213 | 3190474 | 1893489 | 1404944 | 167920 | 887231 | 125481 | 220334 |
| D-(+)-Malic acid | 2818 | 2893 | 11886 | 1493 | 1335 | 403286 | 227842 | 508810 | 310501 | 132945 | 127640 | 169677 | 235348 | 106801 |
| NICOTINAMIDE | 9725 | 14150 | 11196 | 10794 | 13016 | 69936 | 16978 | 106099 | 96843 | 63065 | 33682 | 40902 | 14189 | 31858 |
| Adenine | 6384 | 6471 | 10434 | 6272 | 6448 | 202755 | 96135 | 66862 | 240324 | 189478 | 1516 | 96137 | 2283 | 6272 |
| Uridine | 19413 | 20849 | 27677 | 18330 | 32221 | 247672 | 185241 | 373552 | 309160 | 207987 | 620 | 49088 | 943 | 9039 |
| Adenosine | 28599 | 29950 | 49009 | 19490 | 30776 | 192051 | 196126 | 12363 | 129015 | 324527 | 590 | 179548 | 965 | 81338 |
| 2'-Deoxyguanosine | 15435 | 22722 | 32569 | 15402 | 21159 | 441352 | 278258 | 495294 | 319943 | 261498 | 4203 | 13687 | 63629 | 33119 |
| (-)-Riboflavin | 5038 | 10487 | 6853 | 5408 | 15179 | 77179 | 58398 | 136117 | 76204 | 55787 | 10276 | 35942 | 1377 | 154 |
| Guanosine-3',5'-cyclic monophosphate | 721 | 217 | 1183 | 538 | 198 | 71672 | 2947 | 11491 | 157043 | 84638 | 18331 | 36913 | 117 | 320 |
| 3-Methyladenine | 24884 | 30664 | 38874 | 21069 | 16127 | 172995 | 71965 | 208619 | 172698 | 115146 | 10245 | 94460 | 4991 | 6373 |
